# A novel Bayesian method for dual-agent Phase I dose-escalation studies using penalized D-optimality

**DOI:** 10.1186/1745-6215-14-S1-P41

**Published:** 2013-11-29

**Authors:** Graham Wheeler

**Affiliations:** 1 MRC Biostatistics Unit, Cambridge, UK

## 

In oncology, there is increasing interest in studying combinations of drugs to improve treatment efficacy and/or reduce harmful side-effects. Dual-agent Phase I clinical trials are primarily concerned with drug safety, with the aim to discover a maximum tolerated combination dose via dose-escalation; small cohorts of patients are given set doses of both drugs and monitored to see if any particular toxic reactions occur. Whether to escalate, de-escalate or maintain the current dose for either drug for subsequent cohorts is based on the number and severity of observed toxic reactions, and a decision rule.

We propose a novel Bayesian phase I trial design for the study of two chemotherapeutic agents in combination based on a penalized D-optimality criterion. Patients are allocated to dose combinations that provide the most information about the two agents in combination and their interactive behaviour, subject to a penalty function that doses patients at combinations with a probability of toxicity close to some desired target level. Such a criterion compromises between population gain (maximizing information) and patient gain (treating each patient at a maximally tolerable combination).

We show how the design can be applied to a dual-agent Phase I dose-escalation study of Paclitaxel and an Aurora Kinase Inhibitor in combination. Our simulation studies show that the method provides accurate and tolerable recommendations for further clinical testing and has the ability to outperform other proposed methods.

